# Ten Years of Neonatal Intensive Care Adaption to the Infants’ Needs: Implementation of a Family-Centered Care Model with Single-Family Rooms in Norway

**DOI:** 10.3390/ijerph19105917

**Published:** 2022-05-13

**Authors:** Lene Tandle Lyngstad, Flore Le Marechal, Birgitte Lenes Ekeberg, Krzysztof Hochnowski, Mariann Hval, Bente Silnes Tandberg

**Affiliations:** Department of Paediatric and Adolescent Medicine, Drammen Hospital, Vestre Viken Hospital Trust, Dronninggata 28, 3004 Drammen, Norway; flokun@vestreviken.no (F.L.M.); sbekeb@ous-hf.no (B.L.E.); krzysztof.hochnowski@vestreviken.no (K.H.); mariann.hval@vestreviken.no (M.H.)

**Keywords:** neonatal intensive care unit, family-centered care, single family room, parents, cultural change, implementation, Plan-Do-Study-Act (PDSA) cycle

## Abstract

Ten years ago, the Neonatal intensive care unit in Drammen, Norway, implemented Single-Family Rooms (SFR), replacing the traditional open bay (OB) unit. Welcoming parents to stay together with their infant 24 h per day, seven days per week, was both challenging and inspiring. The aim of this paper is to describe the implementation of SFR and how they have contributed to a cultural change among the interprofessional staff. Parents want to participate in infant care, but to do so, they need information and supervision from nurses, as well as emotional support. Although SFR protect infants and provide private accommodation for parents, nurses may feel isolated and lack peer support. Our paper describes how we managed to systematically reorganize the nurse’s workflow by using a Plan-Do-Study-Act (PDSA) cycle approach. Significant milestones are identified, and the implementation processes are displayed. The continuous parental presence has changed the way we perceive the family as a care recipient and how we involve the parents in daily care. We provide visions for the future with further developments of care adapted to infants’ needs by providing neonatal intensive care with parents as equal partners.

## 1. Introduction

A Neonatal intensive care unit (NICU) with single-family rooms (SFR) opened at Drammen Hospital in 2012 as a replacement for the traditional open bay (OB) unit. The implementation was thoroughly planned and led by the NICU management. However, in retrospect, we did not anticipate the extent to which the new physical facilities would influence our ways of working. Welcoming parents to stay with their infant 24 h per day, seven days per week, was both challenging and inspiring.

Family-centered care (FCC) is increasingly perceived as an important part of neonatal intensive care. The philosophy of FCC is defined by four core elements, Respect, information sharing, participation, and collaboration (Institute for Patient-and Family^®^, Northern, VA, USA), and is generally accepted as the working platform for NICU professionals. However, the degree of implementation of FCC may differ significantly between units [1,2,3]. A NICU culture may build upon values according to the FCC philosophy independently of the physical facilities; however, the design of SFR provides a more beneficial, stress-reduced environment for infants and the opportunity for parents to stay with their infant in more private accommodations during the entire hospital stay.

The starting point for planning our SFR unit was the reorganization of preterm infant patient flows within the health region, which led to a 50% increase in patient volume. The unit expanded from 12 to 17 beds and currently receives 450 admissions per year from four hospitals. The unit provides care for all infants born with a postmenstrual age (PMA) from 28 weeks within their hospital referral area. Preterm infants younger than 28 weeks are born at the university hospital, and they are transferred to our unit when they are in a stable condition. The expansion led to a large economic investment of NOK 46 million (corresponding to about 6 million US dollars). At the time, there was a lack of clinical evidence of the positive impact on the infant and family in SFR design. Therefore, the management team, led by the senior consultant, had to convince the hospital board and politicians to allow such a large investment. Arguments in favor of the SFR were based on previous experiences of increased parental presence when we placed adult beds beside the incubators in the open bay. The main reasons for promoting and building an SFR unit was the potentially severe consequences of separating infants from mothers, as well as the acknowledgement of infants’ fundamental human right to be close to their parents [4]. When parents are allowed unrestricted access to their infant, they can participate more actively in shared decision making at an informed and competent level, based on their knowledge of their infant. Parents were to be perceived as an acknowledged partner in their infant’s treatment and care and not as visitors.

A careful overall description of the medical treatment and care and family support provided in SFR is limited in previous research reports [5]. Thus, our 10 years of experience with SFR may be helpful and inspiring to others. Although the implementation of SFR was performed according to the Plan-Do-Study-Act (PDSA) framework, this paper does not provide a complete systematic evaluation, but rather, it provides a description and discussion of some significant elements in the implementation process.

The aim of this paper is to describe the implementation of SFR and how it has contributed to change our mindset and ways of working.

## 2. Milestones

In retrospect, we wish to identify important milestones towards care and medical treatment guided by the needs of preterm and ill infants. During the early years of the new millennium, a renewed focus on attachment emerged, which yielded new knowledge on how infants’ early attachment experiences shape their understanding of the world and themselves. A great number of studies have documented the effect of various programs aimed at sensitizing parents to their preterm infant’s signals, thereby increasing attachment and interaction [6,7,8].

The initial significant milestone was reached in 2004, when the NICU in Drammen became the first Norwegian unit to implement an early intervention program with a theoretical framework based on the Vermont model [7,9]. An interprofessional project group [10] led by a child psychologist developed a program that was customized for our unit. A local guide for the interpretation of preterm infants’ behaviour and the supervision of parents in supporting their infant was developed (unpublished). The program generated insights into how well infants communicate with their parents when given the opportunity.

The next milestone was increased knowledge of the benefits of skin-to-skin care [11,12,13]. The parents were encouraged to care for their infant skin-to-skin when they visited the unit. Comfortable chairs were provided to the parents, but there was still limited space and a lack of privacy. In 2008, a group of nurses travelled to Uppsala University Hospital in Sweden to learn how to instruct parents in carrying their infant in a kangaroo shawl, but no systematic implementation took place at the time. One year later, we shifted to a more family-friendly philosophy by implementing a structured visitation program for siblings [14]. The intention was to ensure that siblings were welcomed to the unit by the nursing staff with age-appropriate communication and interaction, characterized by knowledge and adjusted to each sibling’s developmental level. Another important milestone was reached in 2010, when the unit made space for an adult bed beside the infants’ cot or incubator. We wanted to provide a place for parents to rest, feed their infant, and have skin-to-skin contact (SSC). Our immediate experience was that parents who had access to a bed stayed in the unit for longer periods of time during the day and even during the night if allowed by the nurses.

## 3. A Minimal Handling Strategy

**Figure 1:**
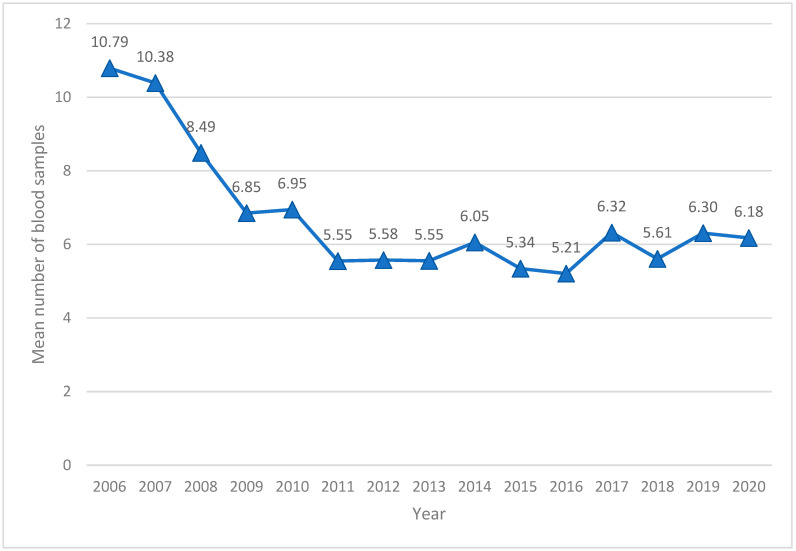
Mean number of blood samples per patient stay per year.

The unit has practiced a ‘minimal handling’ strategy since early 2000. The concept of minimal handling is often referred to, but there is no specific definition of the term in the literature. A minimal handling strategy strives to avoid ‘overdiagnoses’ and ‘overtreatment’, which entails that there are no routine tests or procedures performed without critical reflection. The physicians are instructed to ask three questions before they perform a diagnostic test: Is it likely that the result will change the current treatment or clinical judgement of the infant? Do the parents or neonatologist want to know the result of the test? Does the result justify the risk of complications? The number of diagnostic tests (blood samples/venipuncture, X-rays, echocardiography/ultrasound) performed per patient are registered and used actively as an indicator of quality. In addition, we focus on ordering the right samples. Packages of tests are avoided as much as possible, as the neonatologists’ aim is to target the problem and reflect upon the indication for sampling. The benefit is a reduced number of punctures, with a decreased amount of stress and pain for the infant, less blood volume drawn, and reduced incidents of iatrogenic anemia in preterm infants (Figure 1). In 2008, the unit improved the minimal handling strategy further. The blood sample procedure was revised from the current practice, with heel pricks performed by laboratory staff on fixed routine rounds, to scalp venous punctures performed by the nurses and adjusted to the infants’ rhythm [15].

## 4. From Open Bay to Single-Family Room

**Figure 2:**
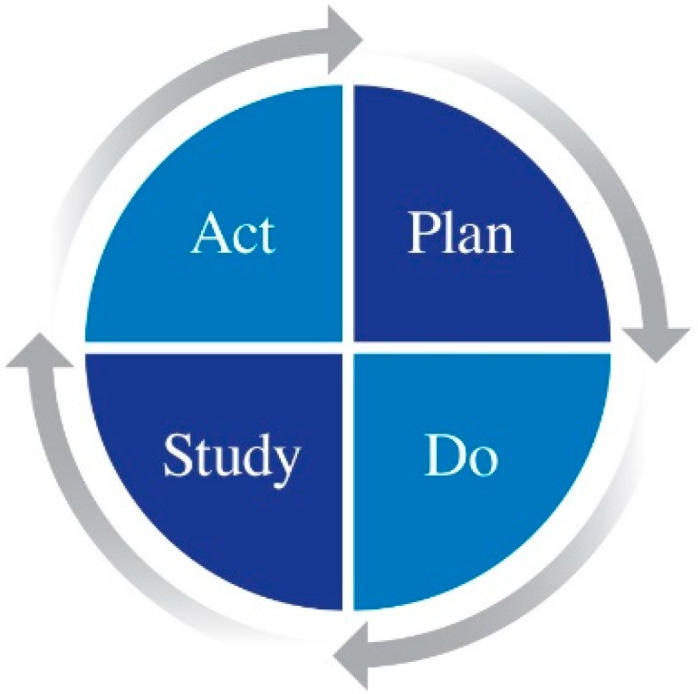
PDSA cycle (The W. Edwards Deming Institute^®^, Ketchum, ID, USA).

Implementation of the new unit with an SFR design was conducted via a stepwise approach based on a PDSA framework (Figure 2).

This structured framework supports practice change as follows: (1) plan a desired change (plan), (2) execute the elements of the change (do), (3) study the change by measured outcomes (study), and then (4) adjust based on the acquired information (act) to begin another cycle [16] (Table 1).

The early preparations included establishing an interprofessional steering group, acquiring financial funding, and planning the SFR unit design. We actively searched for expert knowledge and experience. The management team visited two Swedish SFR NICUs (Karolinska University Hospital and Uppsala University Hospital) in 2010 and was inspired and supervised in the first important implementation phase. The next step was the preparation of the interprofessional staff for the upcoming change in physical environment, work routines, and continuous parental presence.

The steering group consisted of the head nurse, clinical nurse specialists, and the senior consultant neonatologist. A theoretical framework on FCC was designed for the NICU staff and mandated for study before the opening of the SFR unit in April 2012. In addition, repeated theoretical lectures and practical training with a focus on procedures related to providing safe SSC, support for infant–parent closeness, and parental involvement were conducted. Midwives provided theoretical lectures on the needs of postpartum mothers. Furthermore, nursing staff were given lectures and practical training about communication and interaction with parents. An overall objective of the unit with a detailed description of each goal, e.g., ‘80% of the admitted preterm infants shall be cared for skin-to-skin with the mother or father within 24 h after birth’, was developed. All clinical procedures were revised according to FCC values [17,18], and new tools were developed and pilot-tested before being incorporated into the SFR unit. An example of these tools was a registration scheme for the daily duration of SSC.

Nevertheless, at the time, the focus was mostly placed on how to provide information to parents upon hospital admission and how to supervise them in daily care and SCC; less emphasis was placed on identifying emotional distress and exhaustion among parents and providing them with adequate emotional support. To minimize separation and support closeness after birth, we collaborated with the maternity ward to provide SSC for the first two hours postpartum for infants born between 32–34 weeks of gestational age. The simulation training sessions together with midwives and gynecologists from the maternity ward were carried out one year before opening the SFR unit.

## 5. Moving into the SFR Unit

The new SFR unit opened in April 2012. With 15 SFR and no OB areas, this unit represents a new era in Norwegian NICU design. The unit consists of eight intensive care rooms for infants in need of ventilation support and seven family rooms for medically stable infants. The intensive care rooms provide two separate areas: one infant area, with a space for an incubator and technical equipment, and one parent area, with beds and bathroom accommodations. During the daytime, there is no physical separation between the infant and parent areas. The equipment is mounted on flexible arms to allow for the safe transfer of infants from the incubator to the parents’ bed without disconnection. Flexible folding doors enclose the sleeping area and ensure privacy for parents during the night. The family rooms are designed with the infants’ bed close to the parents under the expectation that the parents gradually take over infant care. Healthy siblings are welcome to visit and stay overnight without limitations. Mothers are required to sleep at the obstetric ward on the first night after birth in the interest of their own maternity health and safety, but they are allowed to be close to their infant for the rest of the time. Fathers are admitted to the unit immediately after birth. All meals are provided free of charge for both parents.

At the time of its opening, the unit had approximately 46 nursing positions entailing a variety of educational levels and experiences: registered nurses with less than one year of experience; nurses with a postgraduate education in intensive care; pediatric and neonatal nurses with over 20 years of work experience within NICUs. The SFR unit has five fully trained lactation support providers, six neonatologists, two midwives and a part-time clinical psychologist. In addition, services are provided by physiotherapists, social workers, dietists, and spiritual providers.

After the opening of the SFR unit, the steering group targeted practical and cultural change by using ‘changing agents’ [19]. A team of experienced nurses organized and supervised the nursing staff. In the first year of implementation, matters of concern were discussed in short daily meetings during day-, evening- and nightshifts. The expert nursing team has had a great impact on the daily organization of the unit, adapting new procedures and ways of working with parents in SFR. This made a crucial contribution to cultural change in the unit, together with the present management, which communicates the unit’s goals in a clear and visible way. The medical rounds were changed from the traditional practice of the health-care team having discussions about the infants’ treatment and care in a separate room to having these discussions in the patient rooms. The neonatologists involve the parents in the discussions before making final medical decisions. The amount of daily SSC, breast feeding, and parental involvement are reported during the medical rounds.

The physical workspace challenged the NICU staff in different ways [20]. The unit was designed with SFR on both sides of a hallway at each end, with two workstations in the middle. The infants are monitored with wireless equipment that allows nurses to receive alerts on mobile phones. This makes it possible for the nurses to maintain responsibility for 2–3 infants and their families in each shift. The fact that the nurses are needed in several rooms simultaneously is a source of exhaustion and frustration for both nurses and parents. The nurses have struggled to organize their shift and have experienced caring for infants and parents to be more demanding and time consuming in SFR. Some parents in intensive care rooms have felt insecure and lonely and experience an overwhelming responsibility for their infant’s health and well-being when nurses are ‘out of sight’. Others have felt that they could be a ‘real family’ when nurses are not present in the room. To permit nurses to stay in the rooms for longer periods without interruptions, e.g., alerts requiring their presence in other rooms, the nurses have been reorganized into small teams during each shift.

Parental presence for 24 h per day, seven days per week, is viewed as supporting natural biological processes. Observing undisturbed parent–infant interactions has contributed to increased understanding about how bonding emerges by avoiding separation. The increased duration of SSC in SFR contributes to positive parental hormonal responses [21,22,23], but other factors related to parental presence may be equally important. According to Flacking et al. [3], parents claim that eye contact, touching, smelling, and breastfeeding are essential for feeling emotionally close to their preterm infant. Early and prolonged SSC is beneficial for hospitalized preterm infants [24]. The SFR design facilitates SCC [25]. A research study was conducted in the unit on whether diaper change is less stressful for preterm infants when performed skin-to-skin with the mother. The results showed that 75% of the preterm infants were less stressed when their diaper was changed skin-to-skin. The unit procedure was thereby changed to reduce stress during diaper change [26]. The implementation of SSC procedures occurred in 2012, based on theoretical lectures for the NICU staff, written information to parents, available demonstration videos and practical supervision. The nurses supervised the parents to hold the infant SSC in two sessions with a duration of a minimum three to four hours throughout the day, as well as during night-time. When parents asked the staff to provide information about the ideal amount of SSC hours, they were advised to hold their infant as much as possible. Parents were asked to register SSC in a written journal, whereas the neonatologists asked the parents to report on the total amount of daily SSC on medical rounds.

Soon after the opening, we observed that fathers were increasingly present and involved in daily care, suggesting that the SFR facilities gave birth to a more active role. The fathers’ continuous presence revealed to us the importance of partnership and shared, equal experiences for NICU parents. The steering group realized soon after the unit’s opening that the social benefits for fathers after preterm birth were not adjusted towards SFR NICUs. Together with the Norwegian ombudsperson for children, the steering group lobbied the government to increase fathers’ rights, which led to extended social benefits and adaptations to new ways of working with the NICU families.

Parents were empowered as caretakers, and the family perspective was extended. Even though the NICU had already implemented a visiting program for siblings, the new SFR unit increased opportunities for siblings to be present and to stay overnight if they wished. The hospitalization of infants after birth influences parents’ psychological well-being [27,28] and the entire family dynamic, including siblings [29]. We came to view the siblings’ presence to be very important for families during long hospital stays, especially during holidays.

We encouraged parents to invite extended family members and close friends to visit, as the SFR enables families to have visitors in their private space. For families with an infant in palliative care, it is considered important to be able to share memories of the dying infant with grandparents and close friends [30,31]. The SFR design makes it possible for families to involve grandparents and close friends in the infants’ daily care, including SSC, to give the parents time to rest and the option of leaving the hospital for shorter periods of time. For families with chronically ill infants or families with a single parent, the emotional and practical effort can be burdensome, especially if the family is staying in the hospital for months [32].

Another aspect of parental presence and SFR is the possibility of monitoring at-risk families and helping parents cope with their new situation [33]. Preterm infants have additional vulnerabilities and an increased risk for ill-treatment within their families. Child maltreatment is a global concern, and NICU nurses working in SFR are ideally placed to identify families at risk of child neglect or abuse and contribute to their protection [33]. The 24/7 parental presence enables the nurses to supervise the parents thoroughly during hospitalization. In cases in which parents need extended supervision after discharge, or their infant needs protection, the unit cooperates with child protection services. Early interventions with at-risk parents may strengthen parent–infant bonding and prevent dysfunctional families. During the 10 years the SFR unit has been in operation, we have been working continuously with at-risk families, and we perceive this to be an important and necessary aspect of NICU care. Thus, it requires adequate multidisciplinary theoretical education in child abuse, supervision of at-risk parents and cooperation with hospital social workers, the maternity ward, and child protection services.

## 6. Evaluation and Adjustment

### 6.1. More Individualized Infant and Family Care

As part of the minimal handling strategy, reducing prolonged and procedural pain has been increasingly prioritized in our unit. An interprofessional quality improvement (QI) project for pain management in preterm and ill-term infants was conducted in the unit in 2017. The project entailed the implementation of the COMFORTneo pain assessment scale, guidelines for assessment and management of pain and stress, and increased parental involvement in painful procedures [34]. One year after the QI project, 89% of the COMFORTneo assessments were performed according to the implemented guidelines. The staff use a flowchart to assess, treat, and reassess the infants’ pain and stress. In line with previous studies, we found that parents want to be involved in their infant’s pain management if they are allowed [35]. During the OI project, parental involvement in non-pharmacological interventions increased from 50.3% before to 82.3% after the implementation. We argue that the success of the QI project was attributable to the systematic use of flow charts, guidelines for pain management, interprofessional collaboration and that the team management actively used experiences gained from the implementation of the SFR unit a few years before.

In retrospect, we had an expectation of increased breastfeeding rates after opening the SFR unit due to the opportunity for mothers to stay close to their infant. However, breastfeeding rates upon discharge declined one year after the unit’s opening. Breastfeeding a preterm infant requires competent breastfeeding support to mothers, both practically and emotionally. To provide such support, nurses must possess knowledge on how preterm infants develop necessary breastfeeding skills [36]. Even though the nursing staff had a long tradition of promoting early breastfeeding and the unit had several lactation consultants, we were not able to sustain satisfactory guidance to the families regarding breastfeeding. It seems like the nurses experienced the change from OB to SFR facilities as an overload, in that their focus shifted to adapting to new ways of working and promoting SSC at the expense of supporting early breastfeeding attempts. In addition, mothers in SFR miss out on the learning possibilities of peer-to-peer support from other mothers. Due to the established tradition at the unit of continuously benchmarking quality, we were able to detect changes in breastfeeding rates over time and to regain focus on breastfeeding when needed. Nevertheless, evidence shows that preterm infants have higher exclusive breastfeeding rates in SFR compared to OB units [37,38].

We have, over recent years, implemented several strategies to support breastfeeding, like breastfeeding rounds once per week with preferably both parents present. The aim of the breastfeeding rounds is to retain a systematic overview of the individual infant–mother breastfeeding dyad. Individual guidance and support are provided by dedicated nurses. The sessions focus on practical and emotional support for families, as well as the development of a plan for the coming days based on the infants’ and mothers’ needs to ensure progression. Parents, regardless of nutrition and feeding type, are invited to be more involved in their infants’ progress from tube feeding to exclusive oral feeding. We have come to learn that parents can be enabled to prepare and perform tube feeding shortly after admission. Upon systematic and safe supervision, most parents appreciate to be involved. The early feeding responsibility provides more natural access to observing and getting to know the infants’ feeding signals. We have experienced that, in recent years, involving fathers in the breastfeeding support sessions is beneficial and can contribute to supporting mothers’ breastfeeding [37,39].

### 6.2. Psychological Well-Being for Parents

Preterm births or births of critically ill infants followed by a NICU admission impose emotional strain on parents [40]. We have previously reported that mothers in SFR have a lower risk of depression and lower parental stress during hospitalization compared to traditional OB units [5,41]. A correlation between parental satisfaction and FCC practices, such as emotional support [5,42,43], has been reported. It has been suggested that variations in NICU environments and care practices may explain the difference in maternal depression rates [44,45]. When parents are engaged in daily care, they experience well-being and a more profound feeling of emotional closeness with their infant [3,46]. Nevertheless, a study conducted in the unit by a psychologist and colleagues during the second year after opening reported that some parents felt pressured to care for their infant skin-to-skin and felt more isolated and solitary in SFR. The parents struggled to balance daily life with demands from the staff concerning presence and involvement in their infant’s care [47]. The advised hours for SSC were thereby adjusted and limited to 6–8 h per day. The parents’ need for uninterrupted sleep during the night was acknowledged and supported by the nursing staff, and the parents were advised to leave the unit once per day for a change in scenery. In the same manner as our monitoring of clinical variables as indicators of quality, we want to highlight the necessity for systematic critical reflection upon how care practices may influence infants and their family.

### 6.3. Change in Roles

The SFR design with continuous parental presence provides privacy and shielded space for practical supervision and psychosocial support to parents. Supervision starts the first day after admission and continues during the entire hospital stay. The nursing staff have a dual role: They care for infants, who often have complex medical needs, as well as supporting parents. Parental presence can be challenging for NICU nurses in SFR, since most parents are experiencing an emotional burden depending on their infant’s health, the mother’s health, the family’s socioeconomic status, and the history of individual parents [48]. The role and demands are changed from traditional task-oriented to a relationship-based approach [49]. SFR require a change in mindset that may be challenging for nurses and neonatologists with prior work experience in OB NICUs, where they had a more paternal role as experts [50]. Their new role may seem less strictly defined when they share responsibility with parents [51]. When nurses evaluated their own performance of FCC in NICU clinical practice, they rated their ability to give emotional support as low [52]. With parents continuously present, there are many opportunities to divulge information and provide guidance and support. However, the SFR unit can also limit parental access to nurses, which makes them vulnerable in specific parent–nurse interactions during each shift.

We observed that involving psychologists in the direct care of families on a daily basis was useful [53,54]. In addition to working closely with parents, the psychologist supervises the nurses in subjects such as the emotional needs of infants and parents, as well as communication with parents. Another area of improvement was the inclusion of the psychologist in the support of parent–neonatologist communication. Every other week, the parents have a private meeting with the neonatologist, supported by the psychologist. The intention is to inform the parents about their infant’s current and expected future situation, as well as to answer questions from parents. However, the psychologist felt that several parents did not understand or struggled to accept the information they were given due to its scope and complexity or had difficulty comprehending the medical terminology used by the neonatologist. The psychologist supports the parents during the meeting by asking questions to the neonatologist on behalf of the parents as well as interpreting the information in a clearer and more straightforward manner. If the parents receive negative information about their infant’s health, the psychologist is able to give them support and comfort during and after the meeting.

### 6.4. Follow up and Outpatients’ Care

In recent years, several NICUs have developed early discharge solutions with extended tube feeding at home, either with advanced home hospital follow up or ambulatory nursing [55]. In the last phase of hospitalization, the infant no longer needs monitoring or medical equipment. The focus is instead on successful feeding and daily care to ensure a safe transition to home care. Due to our experiences with competent families, we have started an early discharge project in which families with healthy preterm infants are provided a digital home follow up (DigiHopp). The purpose of this method is to continue FCC by offering families in the final phase support in their natural home environment. In such care, infants learn to feed at their own pace, and families can adapt to daily life at home in a gentler and more fluid way, without further separation from siblings. Currently, no systematic evaluation of the DigiHopp project has been performed; however, our one-year experience has included mostly positive reports from parents, and the solution seems safe with no adverse events. We perceive DigiHopp as a natural extension of FCC and are therefore planning for its expansion to several patient groups in the future.

## 7. Vision for the Future—Where Do We Go from Here?

**Figure 3:**
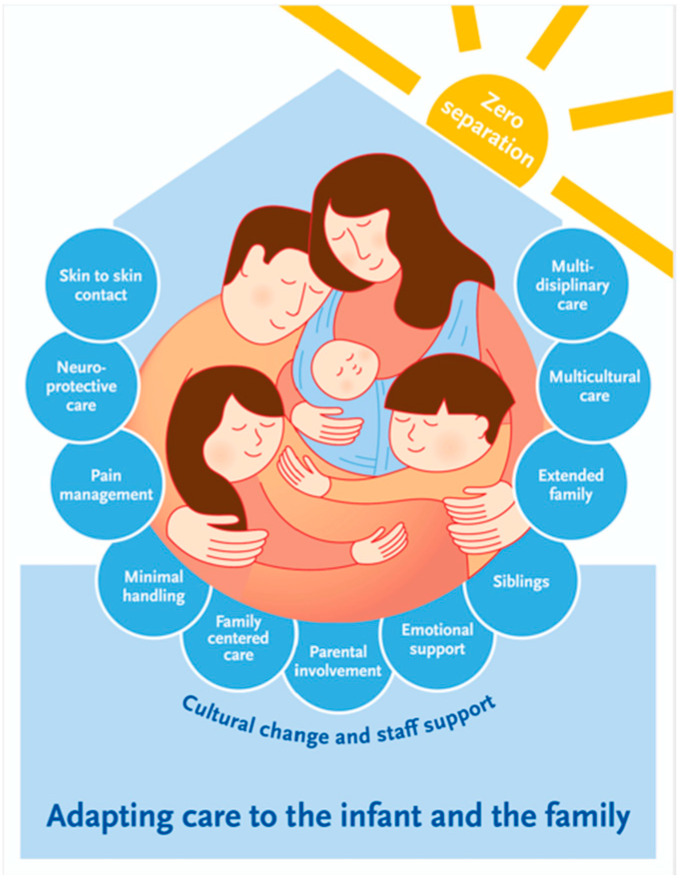
Elements of providing care adapting to the infants needs in a Single-Family Room NICU.

In this paper, we have displayed our process towards FCC in SFR and discussed some main elements which we perceive as fundamental in establishing treatment and care adapted to the infant’s needs (Figure 3).

The SFR unit requires new competence and a change in how we work with parents as caregivers. Further research is needed to generate a more in-depth understanding of parent–staff interactions during SFR care [53,56,57]. Further knowledge about what happens in the SFR and whether parents naturally provide positive stimulation to their infant when they are present is presently lacking. There are still considerable variations in the content, intensity, setting, and degree of parental involvement [58,59], and much remains to be done to integrate parents and optimize the care of preterm and critically ill infants. In the future, we plan for further developments of neuro-protective care adapting to the infant’s need, decreased stress and pain, and increased positive stimulation [60]. We aim at positioning parents as a strong partner in the interprofessional team. The involvement of parents in medical rounds is one way [61]. Increased parental involvement in stressful and painful procedures is another [62]. Several studies have reported that parents have a pain- and stress-reducing effect on their preterm and critically ill infants, but change in practice in this area is lacking [63]. To address the feeding of infants, we aim for a more structural and initialized strategy of feeding readiness, in which infants’ individual behavioral and physiological signals will be acknowledged and interpreted by their mother and by nurses in an equal partnership [64]. We seek to develop new digital information platforms, including decision support, with the purpose of sharing knowledge with parents. The possibilities are infinite in the area of neonatology, but it requires funding, social benefits for parents, and a change in attitudes. Even though the focus in neonatology has shifted from increased survival to continuous improvement in long-term outcomes, infants are still separated from their parents in many NICUs around the world. A continuous development of knowledge and advancement in medical technology, on the one hand, and further development of NICUs that recognizes the whole family as care recipients, on the other, are two important pathways for improvements in neonatology in the future.

## 8. Limitations

Parental presence and involvement in care are more complex and demanding for both the parents and the staff when the infants need high-intensive treatment and care. It may be seen as a limitation for the paper, both in implementing SFR and parental involvement in daily care, that the infant population are overall healthy and not critically ill. Even though this is not a scientific paper, we argue that the careful and honest description of the process and our experiences could be of interest, since SFR is recommended as the NICU gold standard, and similar descriptions are absent [5,38]. Norwegian parents are economically privileged and have one year maternity leave, as well as additional rights after preterm birth for both parents. We fully acknowledge that parental presence in NICUs is dependent on the social benefits for parents after childbirth.

## 9. Conclusions

SFR design prevents infant–parent separation and offers a stress-reduced environment for the infant. In order to support parents and increase parental involvement, a thorough structural and cultural change is required. Parents must be included as both caregivers and care recipients. To be able to change their ways of working and truly consider parents as equal partners, the interprofessional team needs supervision and support. The SFR implementation is comprehensive and requires planning, structure, and strong leadership. Welcoming parents into our unit 24/7 has been a game changer for us to develop treatment and care adapted to the individual infants and families’ needs.

## Figures and Tables

**Table 1:** Quality improvement project: a stepwise approach.

Steps	Description
1. Assessment of the problem	A growing understanding of how the maternal separation negatively impacts the preterm infant’s brain and attachment to the parents. Founded in NICU management Establishment of an interprofessional steering group
2. Literature review	Decision on the Single-Family Room unit framework and common set of unit values
3. Development of an implementation strategy	PDSA cycle as a QI framework Development of comprehensive material for staff education Development of procedures for skin-to-skin contact and parental involvement
4. Education of the NICU staff	Repetitive theoretical lectures during 2011 Practical training in communication Practical training in kangaroo admissions together with the maternity ward
5. Implementation of new schemas/tools and guidelines	Systematic use of procedures and tools Daily supervision, reports, and discussions during medical rounds on quality indicators
6. Monitoring of quality improvement data	The Norwegian quality registry for infants
7. Continually improvement	Revision of procedures Changes in medical rounds The unit psychologist participates routinely in the parents–neonatologist meetings

NICU: Neonatal intensive care unit; PDSA: Plan-Do-Study-Act; QI: Quality improvement.
